# Readability of paediatric participant information leaflets in research studies

**DOI:** 10.1038/s41390-025-03943-z

**Published:** 2025-02-22

**Authors:** Cian P. O’Halloran, Abhishek Agarwal, Daniel B. Hawcutt, Louise Oni, James Moss

**Affiliations:** 1 Mersey and West Lancashire Teaching Hospitals NHS Trust, Prescot, UK; 2 NIHR Alder Hey Clinical Research Facility, Alder Hey Children’s NHS Foundation Trust, Liverpool, UK; 3 Department of Women’s and Children’s Health, Institute of Life Course and Medical Sciences, University of Liverpool, Liverpool, UK; 4 Department of Paediatric Nephrology, Alder Hey Children’s NHS Foundation Trust, Liverpool, UK

## Abstract

**Background:**

Information leaflets in research studies should be age-appropriate to be understood, however the formal readability of children’s participant information leaflets (PILs) for research studies has not been assessed.

**Methods:**

A single-centre cross-sectional study assessing paediatric PILs. Six readability tests were applied (Gunning Fog Index (GFI), Simple Measure of Gobbledygook (SMOG), Flesch Kincaid Grade Level (FKGL), Coleman–Liau Index (CLI), Automated Readability Index (ARI) and Flesch Reading Ease score (FRE). Results were compared between age groups, and whether the PIL was from either a commercially sponsored or investigator led study.

**Results:**

191 paediatric PILs were included. Age categories; <10 years (*n* = 65), ≤12 (*n* = 73), ≤15 (*n* = 73) and ≥16 (*n* = 61); were used for analysis. There were 39 commercial PILs and 226 non-commercial PILs. For the ≤10 and ≤12 age bands, all 6 median readability scores exceeded the target age group (thus hard to read, p < 0.005), and there was no difference in readability scores between these two age bands. Four scores from the readability tests were considered age-appropriate in the ≤15 year category, and all median scores were age-appropriate in the ≥16 years age groups. Readability scores for children’s PILs were significantly higher in commercially sponsored versus non-commercial studies (*P* < 0.005).

**Conclusion:**

Improvements are required to make children’s PILs readable for the target audience, particularly in commercially sponsored research studies.

**Impact:**

- Paediatric participant information leaflets may not be readable in research studies, especially in younger age groups.
- PILs for children participating in commercially sponsored studies were less readable than non-commercial studies.
- Research teams writing PILs for a paediatric study need to consider the use of readability tools to ensure that the information they are providing is readable by the target audience.

## Introduction

It is well recognised that poor health literacy is associated with suboptimal healthcare outcomes.^1^ Poorly understood healthcare materials can compromise patient engagement and treatment adherence but also increase hospital admissions, use of emergency care, and mortality rates.^2^ Patients with low levels of education, residing in low socioeconomic regions, and long-term health conditions or disabilities are particularly affected.^3,4^ To this end, there is a substantive need to understand how to improve access to medical information.^5,6^

Whilst emerging studies have shed light on the health illiteracy of the adult population, understanding of health literacy in the paediatric population is scarcer, with early suggestions indicating that children from ethnic minority ancestry and living in families of low income are most affected.^7^ Children have a legal and moral right to be able to participate in decisions that affect them. Surveys of children and young people’s healthcare experiences have identified that feedback from children themselves is generally less positive than their parents’ responses, with a third of children in one survey reporting that they did not always understand what staff had said and over a half of children feeling that they were not sufficiently involved in making decisions about their care or treatment.^8^

Participant Information Leaflets (PIL) are provided as a core part of recruiting to research studies, and they enable informed consent/assent for the study.^9^ In children, these are provided to the parents for consent and to the children/young people for assent under the age of 16 years. Although asking young children (those under five) to sign an assent form may not be appropriate, their views towards participating in the study should still be sought.^10^ Up to 43% of adults in the UK are unable to read above the level of a GCSE grade D-G (US school grade/readability score of 6–7).^11^ Our previous work showed the readability of PILs designed for parents was significantly above the recommended reading age, with 81% of PILs classed as difficult to read.^5^

Readability formulas are validated tools, suggested by national frameworks,^12–14^ that are used to evaluate the reading age of a piece of text. These formulas calculate a readability score representing the equivalent reading ability in terms of formal years of schooling. Gunning Fog Index (GFI), Simple Measure of Gobbledygook (SMOG), Flesch Kincaid Grade Level (FKGL), Coleman–Liau Index (CLI), the Automated Readability Index (ARI), and Flesch Reading Ease Score (FRE) are examples of the most extensively used formulas in healthcare settings.^15^ When using different readability formulas, it is suggested that the highest calculated reading age, or an average across the tools, can be used to determine overall readability.^5,6^ The FKGL is the most widely used tool to assess material readability, whilst the SMOG formula has been suggested as the most suited for healthcare applications due to its better consistency of results, higher level of expected comprehension, use of more recent validation criteria for determining reading grade level estimates, and simplicity of use.^6^

The primary aim of this study was to determine the readability of PILs, as part of research studies, that have been designed specifically for children and young people across a range of ages and compare this to the national literacy level expected for their age. Our previous work highlighted that commercially sponsored studies are less accessible to parents than their non-commercial equivalents. Thus, the secondary aim of this study was to compare the readability scores of children’s PILs between commercially sponsored studies (e.g. by pharmaceutical or medical device companies) and non-commercially sponsored studies (e.g. academic or National Health Service (NHS) institutions).

## Method

An online readability tool^16^ was used to analyse PILs of clinical research studies, yielding the GFI, SMOG, FKGL, CLI and ARI readability scores. The same portfolio of research studies was used as our previous work on parent PILs.^5^

### Setting

The study was a single-centre cross-sectional study undertaken at Alder Hey Children’s NHS Foundation Trust Hospital, Liverpool, UK.

### Eligible studies

Eligible studies were obtained using records from the previously published study using parent PILs to allow comparison. The clinical trial portfolio list was obtained on July 4, 2022. It included only studies that were open to recruitment at the time, while studies that were closed to recruitment, even if still open for follow-up data, were excluded from the list.^5^

Neonatal studies were excluded as readability scores would be inappropriate in this age category. Studies were also excluded if the intended age was not specified on the PILs, as we could not draw any conclusions from the target age of these PILs. Most studies had several PILs (e.g. a PIL for those aged 5–8 years of age, 9–11 years of age, 12–15 years of age etc.). This accounts for the high number of PILs vs number of studies. PILs were also excluded if they had a duplicate but almost identical PIL within the same age range for a different cohort of patients in the study. In these circumstances, the investigators (C.O.H/JM) selected one representative PIL for analysis to avoid duplication and not skew the data. Studies were also excluded if the PIL was represented in picture format as this could not be analysed using the online readability tool.^16^

### Ethical approval

As this study only involved a secondary review of PILs with no direct patient involvement, ethical approval was not required. The study was registered with the Trust’s clinical audit department as an evaluation of existing services (reference number 6888).

### Readability software and data collection

Multiple readability tools were used for this study as there is no one gold standard. All text on the PIL was evaluated, including contact data and regulatory information. The subtype of study in terms of commercial or non-commercially sponsored was recorded. The number of characters, number of total words, number of sentences, lexical density, average number of characters per word, average number of syllables per word and average number of words per sentence were recorded together with the Flesch reading ease score (FRE). The FRE is a reading age tool based on the average sentence length and average number of syllables.^17^

### Data interpretation and statistical analysis

Readability scores (apart from FRE) roughly translate to formal years of schooling according to the US academic grade.^18^ For example, a readability score of 7–7.9 should be readable by a grade 7 student in the American system, equivalent to 7 years of schooling, and translating to year 8 in their UK counterparts (i.e. age 12–13). Table 1 translates readability scores into their respective target audience.^19^

**Table 1:** The American school grade system and their associated expected readability age (SMOG, GFI, ARI, FKGL, CLI), translated to the corresponding student age and equivalent UK schooling level.

Age (years)	American School Grade Equivalent	UK School Grade Equivalent	Readability score	FRE scores (0–100)
7–8	Grade 2	Year 3	2	90–100
8–9	Grade 3	Year 4	3	90–100
9–10	Grade 4	Year 5	4	90–100
10–11	Grade 5	Year 6	5	90–100
11–12	Grade 6	Year 7	6	80–90
12–13	Grade 7	Year 8	7	70–80
13–14	Grade 8	Year 9	8	60–70
14–15	Grade 9	Year 10	9	60–70
15–16	Grade 10	Year 11	10	50–60
16–17	Grade 11	Year 12 / Lower 6^th^	11	50–60
17–18	Grade 12	Year 13/ Upper 6^th^	12	50–60

FRE scores are included for comparison.

For this study, PILs were grouped by age, where the upper age limit of the PIL defined what ‘group’ it was analysed in. For example, PILs with an age range of 6–8 years or 6–10 years would be grouped into PILs ≤10 years, whereas a PIL with an age range of 7–12 years would be analysed in both the PIL ≤ 10 years group and the PIL ≤ 12 years group as the age range fell within two groups. Analysis was performed using R Statistical Software (v4.2.0: R Core Team 2022). Data were tested for normality using the Shapiro–Wilks test and non-normally distributed data were assessed for significance using Kruskal–Wallis and Mann–Whitney U tests. A *p*-value of <0.05 was taken as statistically significant.

## Results

### Description of eligible studies

Of the 109 studies previously identified by Nash et al. ^5^ the children’s PILs of 81 studies (74%) were included. Of those excluded, 10 (9.2%) studies did not define the intended age of the PIL, 9 (8.3%) were neonatal studies, 4 (3.7%) were only available in inaccessible picture format and therefore not analysable, 3 (2.8%) were unobtainable, and 1 (0.9%) was for parents only and 1 (0.9%) was for staff members only. Most studies (*n* = 67, 83%) were categorised as non-commercial. 191 children’s PILs were included in the study and analysed for readability. 74 (38.7%) of these were placed into more than one age group, meaning there were 65 PILs in the ≤10 years category, 66 PILs in the ≤12 years, 73 PILs in the ≤15 years, and 61 PILs in the ≥16 years category.

### Readability scores according to each tool

**Fig. 1:**
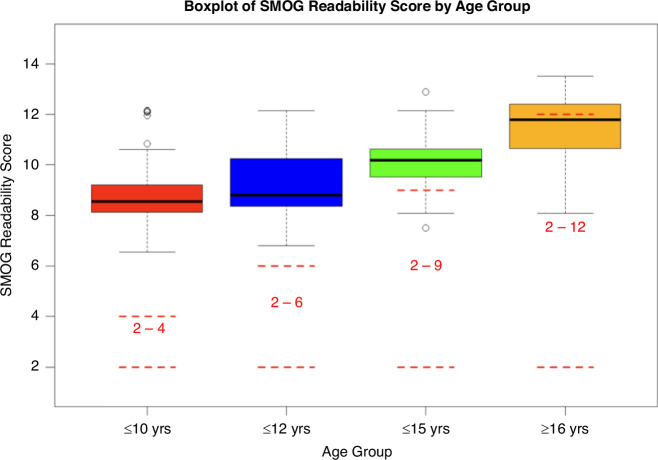
Boxplot of SMOG readability score for paediatric PIL’s assessed within the specified age groups. Number and the dashed lines in red represent the accepted SMOG readability score for that age group.

The Kruskal–Wallis test showed a statistically significant difference between all age groups for the different parameters assessed (*P* < 0.001). Median values for the different age groups of parameters assessed are shown in Table 2. When comparing all groups, there was no statistically significant difference between the ≤10 years and ≤12 years age groups in terms of the readability scores, suggesting they are pitched at a similar audience. However, there was a statistically significant difference between the rest of the readability scores of the PIL age groups as expected. A boxplot of the SMOG scores for each of the children’s PIL age categories is shown in Fig. 1.

**Table 2:** Readability scores of paediatric PILs by six automated tools in different age groups.

Parameter	PIL’s ≤ 10 years, *n* = 65 (median, IQ range)	PIL’s ≤ 12 Years, *n* = 66 (median, IQ range)	PIL’s ≤ 15 years, *n* = 73 (median, IQ range)	PIL’s ≥ 16 years, *n* = 61 (median, IQ range)	*p* value
Upper limit of suggested readability score^a^	4	6	9	12	n/a
GFI	8.42 (7.74–9.48)	8.7 (8.14–10.10)	10.01 (9.41–10.62)	11.75 (10.45–12.876	*P* < 0.005 between all groups except ≤10 years and ≤12 years
CLI	6.84 (6.13–7.79)	7.16 (6.45–8.70)	8.38 (7.60–9.43)	10.17 (9.09–10.78)	*P* < 0.005 between all groups except ≤10 years and ≤12 years
FKGL	7.45 (6.95–8.24)	7.77 (7.10–8.86)	9.07(8.47–9.76)	10.51 (9.36–11.67)	*P* < 0.005 between all groups except ≤10 years and ≤12 years
ARI	5.89 (4.79–6.82)	6.16 (5.18–7.92)	7.90 (6.64–8.86)	9.54 (8.41–10.75)	*P* < 0.005 between all groups except ≤10 years and ≤12 years
SMOG	8.55 (8.12–9.20)	8.81 (8.34–10.24)	10.18 (9.53–10.62)	11.79 (10.64–12.40)	*P* < 0.005 between all groups except ≤10 years and ≤12 years
Lower limit of suggested FRE score	90	70	60	50	n/a
FRE	68.1 (62.63–71.41)	66.88 (59.21–70.32)	60.17 (55.81–63.10)	50.22 (45.31–55.79)	*P* < 0.005 between all groups except ≤10 years and <12 years

*GFI* Gunning fox index, *CLI* Coleman–Liau Index, *FKGL* Flesch Kincaid Grade, *SMOG* Simple Measure of Gobbledygook, *ARI* Automated Readability Index, *FRE* Flesch Reading Ease score, *IQ* Interquartile.

^a^Not applicable to the FRE; Upper limit for readability scores is used as a reference point, as readability scores increase with age and thus should not be above this limit, apart from the FRE, where readability scores decrease with age, and thus should not be below this limit.

### Comparison of the readability of PIL according to commercial and non-commercial subtypes

When comparing readability scores using different tools across the children’s PILs according to whether they were commercial or non-commercially sponsored studies, there was a statistically significant difference between all the measured parameters (Table 3).

**Table 3:** Readability scores of paediatric PILs: Commercial Vs. Non- Commercial studies.

Parameter	Commercial, *n* = 33 (median, IQ range)	Non-commercial, *n* = 158 (median, IQ range)	*p* value
GFI	11.14 (9.48–12.77)	9.77 (8.43–11.02)	*P* < 0.05
CLI	8.81 (7.55–10.74)	8.02 (6.62–9.64)	*P* < 0.05
FKGL	10.11 (8.62–12.01)	8.61 (7.32–9.88)	*P* < 0.05
ARI	8.86 (7.20–11.07)	7.24 (5.56–9.05)	*P* < 0.05
SMOG	10.51 (9.39–12.44)	9.81 (8.55–10.92)	*P* < 0.05
FRE	56.69 (44.20–63.81)	61.82 (54.53–68.54)	*P* < 0.05

*GFI* Gunning fox index, *CLI* Coleman–Liau Index, *FKGL* Flesch Kincaid Grade, *SMOG* Simple Measure of Gobbledygook, *ARI* Automated Readability Index, *FRE* Flesch Reading Ease score, *IQ* Interquartile.

## Discussion

Informed assent is widely recommended as an integral part of paediatric research studies.^10^ Whilst practical guidelines exist to support researchers in the design of patient information materials,^12–14^ there is little evidence to indicate that PILs are understandable to their readers.^5^ The study by Unguru et al., showed that over half of children participating in an oncological research study were unaware that their treatment was considered research, and 86% did not understand the information given to them by their doctor when discussing the trial.^20^ In other studies, less than 50% of children understood the purpose of the study or the study protocol, just over 50% understood the risks involved,^21^ and over a third of children had no recollection of study information.^22^ The aim of this study was to analyse a large cohort of children’s PILs to better understand whether readability is a barrier to inclusive research. The results from this study indicate that the information related to paediatric studies is pitched at a reading level exceeding that of the intended recipient and is aligned with the findings from our previous study evaluating parent PILs.^5^

Our analysis suggests that a child below the age of 10 years is expected to read at the level of a 13–14-year-old. For example, the median SMOG readability score for our less than 10 age group was 8.44, more than double the suggested readability score of 4 for this age group. Adolescents are key drivers of their healthcare outcomes, and it is conceivable that if they need help understanding the information presented to them, they may be less likely to participate or actively engage with a research study.^23^ In this event, parent information leaflets become even more integral, as the child will likely ask their parents for clarification. As previously shown, parent information leaflets are also largely inaccessible to those they are intended for, with only 1 out of 109 parent information leaflets being pitched at an acceptable reading grade (i.e. 11–12 years old, or a readability score of 6).^5^ Applying the same principle to our data and using the SMOG readability score (the score most suited for healthcare applications) then none of our age groups would be considered appropriate as they all had a median SMOG score of 8.55 or above, far above the national literacy suggested readability score of 6 (i.e 11–12 years old).

It is worth highlighting that there was no statistically significant difference in readability scores between those 10 years of age and below and 12 years of age and below. Although there was overlap in the PILs in each group e.g. a PIL intended for those aged 8–12 years old was analysed in both the ≤10 and ≤12 groups, this was also true for other age groups. This suggests that PILs for those 10 years and under, and those 12 years and under, are written to the same reading level. Further, in both age categories, all 6 readability tools suggested these PILs were not age appropriate.

When comparing commercially sponsored and non-commercially sponsored studies, there was a statistically significant difference between all measured parameters. Readability scores were markedly worse in commercially sponsored studies, similar to our previously published study.^5^ However, similar work on readability and understandability of clinical research patient information leaflets and consent forms in the UK and Republic of Ireland did not show a significant difference in readability (FRE, FKGL, GF, SMOG, NDC, Fry, Raygor) or reading age with sponsor types (academic, hospital-based, collaborative group or industry sponsors). They did, however, report that none of the PILs or informed consent forms had the mean reading age of <12 years as recommended by the American Medical Association and use as a benchmark in previous studies.^24^ This calls for a wider review to determine whether there exists a difference in the readability of PILs between commercially and non-commercially sponsored studies to identify areas for improvement.

Our results indicate that paediatric patients in clinical trials receive healthcare information they may not understand before consenting/assenting to trial participation. Further, the results suggest that those participating in commercial studies are at even greater risk of inaccessible information. There is a critical need then, particularly in commercial studies, to adapt practices and produce information leaflets that are more readable to paediatric patients and easier to understand.

Notably, readability calculators come with limitations as they can only analyse the text. They do not show whether writing is interesting or enjoyable, and design and layout is not accounted for – both of which contribute to how easy something is to read.^12^ They do not analyse the font size, punctuation, spacing, bullet points, headers, or any visual aids such as images, graphs, flow diagrams or video links associated with the text provided in the patient information leaflets. For example, the use of a larger font size and pictures were particularly popular amongst children when assessing patient information leaflets.^21,25^ A child’s subjective opinion, and the authors’ objective opinion of whether the child understood the PIL, as previously demonstrated may be a useful addition then to supplement future work assessing the readability of PILs in children.^25^ Further, readability tools have a general assumption that longer words and sentences are more challenging to read, which may not always be accurate. They also do not consider the semantic properties of texts, such as the complexity of ideas, the rhetorical structure, and the overall coherence of the text. Changing a text to improve readability scores does not automatically make a text more understandable.^26,27^ In addition to the limitations mentioned when using each scoring tool in our previous paper^5^ and general readability score interpretation as discussed above, shows that this study does have other limitations, which also include being a single-centre site and the supposition that reading ages in the US and UK schooling systems are equivalent.

Using standardised checklists and guidance for creating patient information sheets could improve readability scores and ensure appropriate informed assent or consent. Health Education England and GenerationR, a national network of Young People’s Advisory Groups (YPAGs) based in the UK, recently produced examples of this checklist and guidance.^12–14^ Involving children in the design of PILs, for example by including both a subjective and objective analysis of ‘understanding’ of materials, may supplement readability tools and existing guidelines, to improve the paediatric assent process in research studies.^25^

## Conclusion

Participant information leaflet design is an important part of conducting any ethical research. This study showed that significant improvements are needed to improve readability scores in PILs designed for children and young people, particularly in commercial studies. Involving children and young people in the design of the study would improve the research’s overall success, reliability, and appropriateness. Using a readability formula, such as the SMOG, will help to ensure the PIL’s meet the reading ability of the participants they are intended for.

The datasets generated during and/or analysed during the current study are available from the corresponding author on reasonable request.
